# Epidermolytic Hyperkeratosis: A Challenging Pathology for Clinical Correlation

**DOI:** 10.4274/balkanmedj.galenos.2019.2019.1.127

**Published:** 2019-08-22

**Authors:** Hala M. El Hanbuli, Mohamed H. Elmahdi, Marwa A. Salem

**Affiliations:** 1 Department of Pathology, Fayoum University Faculty of Medicine, Fayoum, Egypt; 2 Lecturer of Pathology, Fayoum University Faculty of Medicine, Fayoum, Egypt; 3 Clinic of Dermatology, Itsa Central Hospital, Fayoum, Egypt

A 1.5-year-old Egyptian boy was presented with widespread, brownish, hyperkeratotic, and slightly verrucous plaques on the skin, since the age of two months. The patient had no history of blistering or generalized cutaneous redness at or after birth. Moreover, he had no medical problems and showed normal growth and development. No other family member was affected by a similar skin condition.

Upon physical examination, brownish, verrucous plaques and papules in a blaschkoid distribution were found on the trunk, and they were more apparent on the extremities bilaterally (Figure 1a), posterior of the neck (Figure 1b), and in the intertriginous areas. His hair and nails were normal, and general examination of other organ systems was unremarkable. The clinical differentials included linear psoriasis and lichen striatus.

A written informed consent was obtained from the parents of the boy, and two skin punch biopsies from the lesions on his nape and knee were taken. Microscopic examination (Figures 1c, d) revealed typical features of epidermolytic hyperkeratosis.

Epidermolytic hyperkeratosis, a rare occurrence, is one of the minor pathological reaction patterns of the skin first described by Ackerman in 1970 and characterized by hyperkeratosis, hypergranulosis, and epidermolysis (1). Under light microscopy, epidermolysis is observed as various-sized clear empty spaces around keratinocyte nuclei with indistinct cell boundaries and premature excessive formation of keratohyaline granules in the upper spinous and granular layers (2).

The use of the term epidermolytic hyperkeratosis to describe the characteristic histological features of this disorder caused a lot of confusion in the literature. It has been described as the fundamental histopathological feature of bullous congenital ichthyosiform erythroderma as well as an incidental finding in other cutaneous disorders, including melanocytic nevi, basal cell carcinoma, isthmus-catagen cyst, leukoplakia, epidermolytic acanthoma, and normal skin and oral mucosa (3). The final diagnosis of this case was therefore very confusing and could not be reached, unless suitable clinical data were correlated with the pathological findings. Therefore, after collecting the clinical and pathological data, a diagnosis of bilateral systematized verrucous epidermal nevus with epidermolytic hyperkeratosis was made.

Verrucous epidermal nevus is a congenital, non-inflammatory, cutaneous keratinocyte hamartoma. It is manifested as papillomatous papules or plaques and often linear or blaschkoid in distribution. The term systematized “epidermal nevus” is used for the lesions that are bilateral and excessive, also known as nevoid ichthyosis hystrix (4). Histologically, verrucous epidermal nevus shows hyperkeratosis, hypergranulosis, acanthosis, and papillomatosis (5). The difficulty in pathological diagnosis is due to the fact that at least 10 different pathological patterns of epidermal nevi have been described with any lesion possibly revealing more than one histological pattern (6). Epidermolytic hyperkeratosis is one of these uncommon pathological presentations of verrucous epidermal nevus (our case) and found in only 16% of such clinical conditions (7). Few similar cases have been reported (6,8,9); therefore, it is important for dermatologists and pathologists to be aware of such an interesting case.

## Figures and Tables

**Figure 1:**
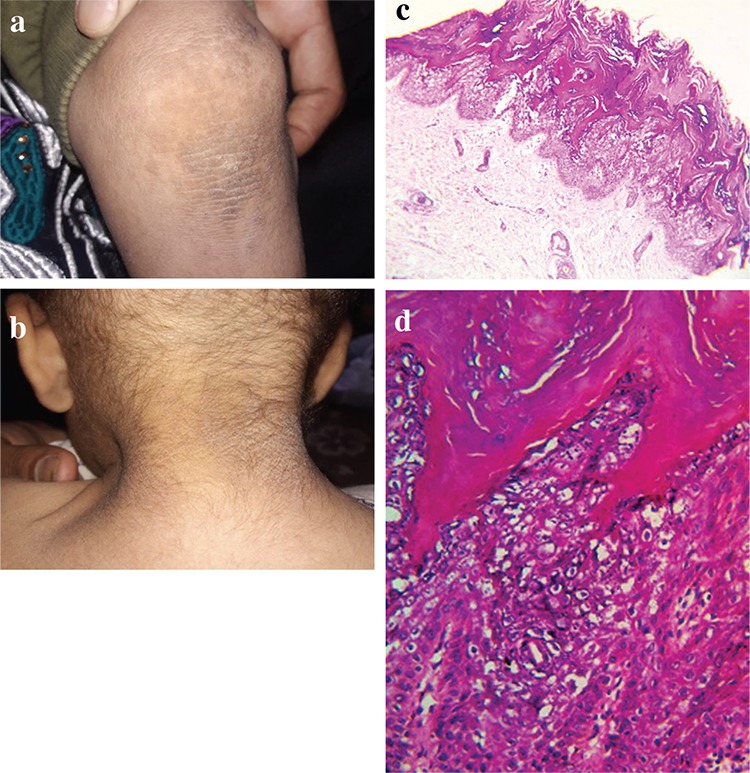
Epidermolytic hyperkeratosis. Dark-brown, verrucous plaques in the extensor of the knee (a) and posterior neck (b). Histopathology of the lesions showing hyperkeratosis, hypergranulosis, acanthosis, papillomatosis, and epidermolytic hyperkeratosis (hematoxylin & eosin, ×100 original magnification); (c) and epidermolysis is observed as various-sized clear spaces around keratinocyte nuclei with premature excessive formation of keratohyaline granules in the upper spinous and granular layers (hematoxylin-eosin, ×400 original magnification) (d).
